# Efficacy and Safety of Corneal Transplantation Using Corneas from Foreign Donors versus Domestic Donors: A Prospective, Randomized, Controlled Trial

**DOI:** 10.1155/2015/178289

**Published:** 2015-01-28

**Authors:** Yingxin Chen, Congling Liao, Minghong Gao, Michael Wellington Belin, Mingwu Wang, Hai Yu, Jing Yu

**Affiliations:** ^1^Department of Ophthalmology, General Hospital of Shenyang Military Area Command, No. 83 Wenhua Road, Shenhe District, Shenyang 110840, China; ^2^Dalian Medical University, 9 West Lvshun South Road, Dalian 116044, China; ^3^Department of Ophthalmology, University of Arizona, Arizona Health Sciences Center, 655 N. Alvernon Way, Suite 108, Tucson, AZ 85711, USA

## Abstract

*Purpose*. To assess the efficacy and safety of corneal transplantation using corneas from foreign donors. *Methods*. One hundred and eight patients needing therapeutic penetrating keratoplasty were randomly divided into 2 groups (54 cases/group): foreign group using foreign donor corneas and domestic group using domestic donor corneas. Clinical outcome and incidence of postoperative complications were compared between groups. *Results*. No significant difference with respect to the therapeutic outcome and postoperative Best Corrected Visual Acuity (BCVA) and neovascularization by final follow-up was observed between the two groups. The graft thickness in the foreign group was statistically higher than the domestic group at 1 month postoperatively, but not at 3, 6, and 12 months postoperatively. Corneal endothelial cell density in the domestic group was statistically higher than in the foreign group at 3, 6, and 12 months postoperatively. Corneal epithelial abnormalities in the foreign group were significantly higher than that in domestic group. The primary graft failure, incidence of graft survival, and postoperative complications such as immunologic rejection, graft infection, and secondary glaucoma were not significantly different between the two groups. *Conclusions*. Corneal transplantations using foreign donor corneas are as effective and safe as those using domestic donor corneas.

## 1. Introduction

Corneal disease is one of the primary eye diseases resulting in blindness, which has become the secondary cause of blindness in China. According to the sample investigation by an investigation team of National Bureau of Statistics of China in 2008, there are vision disability 16,910,000 cases. Of these patients, approximately 4 million of blindness results from corneal disease. Corneal transplantation remains the main method for visual rehabilitation once disease has affected corneal clarity but is dependent on the availability of corneal donor tissue.

An insufficient donor supply is a worldwide problem in corneal transplantation. Due to the effect of religious faith and traditional culture, the corneal donors are lacking in China. There are approximately 3,000 corneal transplants that are performed annually. This number is much lower than the 33,000 corneal transplants performed annually in the United States. “Organ sharing” is one method of ameliorating the insufficiency, and this approach has been used internationally for corneas. Donor corneas transferred from foreign eye banks (foreign donor corneas), enabling more Chinese patients to receive corneal transplants. Meanwhile, foreign donor corneas also bring some challenges. Although the quality of foreign corneas is strictly controlled by eye bank regulations, transportation over long distances may cause unexpected consequences, such as tissue damage or the growth of infectious organisms. Cultural, racial, and environmental differences between the donor and recipient may also cause unpredictable results. Thus, whether foreign donor corneas can well suit for the Chinese population is unknown. Moreover, the efficacy and safety of using foreign donor corneas have not yet been investigated. In this prospective, randomized, controlled study, we investigated and assessed the outcomes of corneal transplantation using foreign and domestic donor corneas.

## 2. Patients and Methods

### 2.1. Patients

The trial was approved by the medical ethics committee of Shenyang General Hospital of PLA, China. All patients provided written informed consent before participating in the study, according to the principles of the Declaration of Helsinki.

All corneal transplantations were performed in the Department of Ophthalmology, Shenyang General Hospital of PLA. All patients received explanation about the origin of the donor corneas and the purpose of the study as well as potential risks of intraoperative or postoperative complications of corneal transplantation. The use of foreign corneas was approved by the Ethical Committee of the Health Department of General Logistics Department of PLA, Beijing, China. With foreign donor corneas, corneas were requested from San Diego Eye Bank in the United States 12 to 16 days before surgery and were usually transported to our hospital on the day before surgery. All foreign donor corneas were obtained from eye banks that follow strict quality criteria. The medical history of the all donors was carefully examined, and serologic tests for human immunodeficiency virus (HIV), hepatitis B and C virus, and human T-cell lymphoma virus I and virus II were performed.

Between February 2011 and December 2012, a total of 108 patients (108 eyes) who need therapeutic penetrating keratoplasty were included using the criteria described below in this prospective, randomized, controlled study. The efficacy and safety of these two groups were compared. The patients were randomly divided into two groups using a successive random number table, with odd numbered patients (foreign group) receiving foreign donor corneas, and even numbered patients (domestic group) receiving domestic donor corneas. Randomization was blinded to the nurses on the inpatient unit, physical therapists, and the patients. The randomization was performed by a research fellow (XX) who was not involved in patient care. Each group contained 54 cases and the characteristics of patients in both groups are summarized in Table 1. In addition, patients were further classified into infectious keratitis group and noninfectious keratitis group according to the type of infection. The reason for dividing patients into two groups was that the recipient beds were noninfectious lesions (e.g., corneal leukoma, corneal degeneration) in noninfectious keratitis group and active inflammation in infectious keratitis group, respectively. These may produce different effects on graft union and postoperative rejects. Under similar donor conditions, comparison of prognosis of different recipients between the two groups will be valuable.

Indications for PKP included (1) bacteria, fungal, and viral keratitis; (2) leukoma caused by trauma or other causes; (3) primary corneal endothelial decompensation after PKP; (4) corneas rejection following keratoplasty; (5) corneal degeneration and dystrophy; (6) bullous keratopathy; (7) keratoconus; (8) iridocorneal endothelial syndrome.

The timing of PKP for patients with infectious keratitis was based on the following classification system: (1) when the infectious ulcer was less than 6 mm in diameter but had not invaded the limbus, we treated with intensive systemic and topical anti-infective drugs. If the infection was not cured and the infectious ulcer increased in size we performed a PKP; (2) when the infectious ulcer was 6–8 mm in diameter and the infection continued to progress during 72 hours of intensive systemic and topical anti-infectious therapy we performed a PKP; (3) when the corneal ulcer was larger than 8 mm or corneal perforation was imminent or had occurred we performed a PKP. The goal of PKP was to (1) repair perforated lesions or ulcer lesions whose corneal perforation was imminent, (2) control infection by peeling off hypopyon, (3) and recover vision by exchange of haze cornea with clear cornea.

### 2.2. Transportation of Foreign Donor Corneas

The corneas were transported as corneoscleral buttons within containers filled with Optisol corneal storage medium (Chiron Vision, Clairmont, CA, USA) and packed in styrene foam with ice packs. The packages were transferred to the airport nearest to the local eye bank, flown to Shenyang Airport, and then transferred to the Cornea Center Eye Bank of Shenyang Military Region by chartered trucking. After delivery, the corneas were carefully evaluated by biomicroscopy and specular microscopy, and only those fulfilling all safety criteria were used. The domestic corneas used in our series were obtained from healthy and fresh human cadaver eyes within 2 hours of death via eyeball extraction surgery. For domestic group, the whole eyeball was preserved in moist chambers maintained at 4°C for <24 hours before use.

### 2.3. Criteria for Patient Selection

To obtain matched pairs in foreign and domestic donor combination, single factor analysis was used to control the clinical outcomes of foreign donor group and domestic donor group using the criteria described below [1]. Paired corneal transplants were chosen using the following criteria: (1) donor and recipients had same original disease; (2) donor and recipients had same immunologic rejection risk status (high risk versus low risk); (3) donor and recipients had similar age, sex, and duration of disease; (4) similar follow-up period; and (5) donor and recipients had same penetrating keratoplasty.

The inclusion criteria were patients whose age ranged from 18 to 70 years; patient with corrected vision acuity no less than light sensation; patients who had bacterial, viral, and fungal corneal ulcers, corneal leukoma, bullous keratopathy, and corneal degeneration; or patients with a history of previous grafting who were considered to have a high risk of immunologic rejection. The exclusion criteria were patients associated with severe lagophthalmos, entropion, and dacryocystitis; end-stage glaucoma and uveitis; severe autoimmune disease; no light perception; not applicable to routine keratoplasty; severe dry eye; not informed of the objective and significance of the study, poor compliance, and over-high expectation.

### 2.4. Assessment of Efficacy and Safety

The following factors were analyzed and compared between the foreign and domestic groups: (i) donor-related factors: (1) donor age, (2) death-to-preservation (D-P) time, (3) preservation-to-operation (P-O) time, (4) endothelial cell density, and (5) microbiologic tests of the preservation medium and corneoscleral rim; (ii) recipient-related factors: (1) age, (2) original disease, (3) degree of neovascularization (0–4+), and (4) duration of disease.

The recipient-related factors were studied because they are reported to influence corneal graft survival [2–5]. Clinical outcomes were compared in terms of best corrected visual acuity (BCVA), corneal thickness, corneal endothelial cell density, corneal graft survival, and the development of postoperative complications, including glaucoma, infection, and immunologic rejection.

### 2.5. Preoperative Treatment

Fungal keratitis patients were treated with fluconazole and natamycin eye drops and intravenous fluconazole. During surgery, 0.02% fluconazole was given intravenously to the anterior chamber and conjunctival sac. Bacterial and viral keratitis patients received topical and systemic antibacterial and viral treatment. Pilocarpine was administered 3–5 times daily 0.5 hour before surgery to avoid the expulsion of intraocular contents. Oral administration of methazolamide 0.5 g once was performed to reduce intraocular pressure and the risk of loss of intraocular contents.

### 2.6. Surgical Procedures

All surgery was performed by the same surgeon (GMH). Generally, penetrating keratoplasties were performed under regional anaesthesia. Retrobulbar block was done with an equal mixture of 2% lidocaine and 0.75% bupivacaine (4 mL), and peribulbar anesthesia was performed when necessary. The donor button was cut with the Barron vacuum trephine (Katena Products, Inc., Denville, New Jersey, USA). The donor buttons were punched from the endothelial side on curved blocks. None of the grafts (6.5–9 mm) extended beyond the limbus. Donor was punched from the endothelial side 0.25–0.5 mm larger than the recipient opening. The recipient bed was prepared to conform closely to the shape of the donor button. A Hessbarg-Barron trephine (Katena Products, Inc., Denville, New Jersey, USA) was used to cut a partial depth, circular incision in the recipient cornea, centered at the geometric center of the cornea. Excision of the recipient corneal button was completed with curved corneal scissors (Storz Instruments Company, San Damis, CA, USA). The corneal transplant was transferred to the host bed and secured with 4 interrupted 10-0 nylon sutures (Ethicon, Johnson & Johnson, Somerville, New Jersey, USA) followed by 12 interrupted or 16 continuous sutures.

Balanced salt solution (Bausch & Lomb, Rochester, NY, USA) was injected to obtain a state anterior chamber. Attention should be paid to the leakage. At the end of PKP, gentamicin 40 mg/0.5 mL and 0.2% fluconazole were injected subconjunctivally for nonfungal keratitis and fungal keratitis, respectively. After surgery, all patients underwent pressure patching.

### 2.7. Postoperative Care

Fungal keratitis patients were treated by fluconazole (once per hour) and natamycin (4 times per day) eye drops and atropine ointment (3 times a day), combined with deproteinized calf blood extract eye gel (3 times a day) and 100 mL of intravenous fluconazole (once per day) from postoperative day 1.

Bacterial keratitis patients were treated by levofloxacin eye drops (twice per hour), ofloxacin eye ointment (3 times a day), tobramycin eye drops (four times per day), and atropine ointment (3 times a day), combined with deproteinized calf blood extract eye gel (3 times a day) and intravenous infusion of cefazolin sodium of a single dose of 1.0 g (twice per day). The operated eyes were wrapped up at the afternoon and continue for 3 days.

Viral keratitis patients were treated with ganciclovir eye drops (4 times per day) and atropine ointment (3 times a day), combined with deproteinized calf blood extract eye gel (3 times a day) and intravenous infusion of a single dose of 250 mL foscarnet sodium. The operated eyes were wrapped up at the afternoon and continue for 3 days.

Noninfectious keratitis patients were treated by levofloxacin eye drops (four times per day), ofloxacin eye ointment (3 times a day), prednisolone acetate 1% eye drops (four-times per day), and atropine ointment (3 times a day). The operated eyes were wrapped up at the afternoon and continue for 3 days.

### 2.8. Follow-Up and Medical Evaluation

Being blinded to the treatment assignments, the surgeon assessed each patient. The patients were followed up at 0.5, 1, 3, 6, 9, and 12 months after the procedures and every 3 months thenceforth. The median follow-up time was 384 days. During that follow-up period, a detailed clinical examination was performed, including best corrected visual acuity (BCVA), clear graft survival, corneal graft thickness, corneal endothelial cell density, neovascularization, rejection episodes, secondary glaucoma, and infection.

### 2.9. Graft Failure

The definition of graft failure, based on the definition used in the Collaborative Corneal Transplantation Studies [2, 6], was a regraft or, in the absence of regraft, a cloudy cornea in which there was loss of central graft clarity sufficient to compromise vision for a minimum of 3 consecutive months.

### 2.10. Graft Rejection

Graft rejection episodes were classified as definite when an endothelial rejection line was present in a previously clear graft and probable when there was inflammation (stromal infiltrate, keratic precipitates, cells in the anterior chamber (AC), or ciliary injection) without an endothelial rejection line in a previously clear graft [7]. Only initial rejection episodes were used in the database for statistical computation.

### 2.11. Ocular Hypertension

A diagnosis of ocular hypertension was recorded in patients who had normal optic nerves, normal visual fields, and applanation tensions of 21 mmHg or greater after surgery or who used pressure lowering medications to lower intraocular pressure. Generally speaking, intraocular pressure can recover after a period of 3–30 days of pressure lowering treatments. Pressure lowering medications can be ceased if the intraocular pressure can stay stable for two weeks.

### 2.12. Glaucoma

A diagnosis of secondary glaucoma was recorded in patients who had a surgical procedure to lower intraocular pressure or who used pressure lowering medications chronically (for 3 or more months) [8].

### 2.13. Infectious Keratitis

The clinical therapeutic effect of infectious keratitis (corneal ulcers) can be classified as cures, improvement, or ineffectiveness. Cures were obtained if there were evidence of visual acuity improvement (VA), inflammation resolution, hypopyon absorption, and ulcer healing, compared to the preoperative level. Corneal ulcers were considered improved if there were evidence of VA no less than preoperative level, partial ulcer healing, and hypopyon no more than preoperative level. The treatment of corneal ulcers were considered ineffective if there were no improvement or even worse in the VA, symptoms and signs, compared to the preoperative level. Therapeutic effectiveness was achieved if corneal ulcers were cured or improved.

### 2.14. Statistical Analysis

Statistical analysis was done by a person not involved in patient care. All statistical analyses were performed with SPSS software (version 13.0; SPSS Inc., Chicago, IL). Results are expressed as means ± standard deviation (SD). Normally distributed data were compared using paired *t* test or Chi-square test. Nonnormally distributed data were compared using Wilcoxon signed-rank test. A generalized estimated equation (GEE) model was used to estimate the corneal endothelial cell density time-course change in the foreign and domestic groups. The incidence of clear grafts in the foreign and domestic groups was compared using the Kaplan-Meyer analysis with the log-rank test to calculate statistical significance. Differences between subgroups in foreign or domestic group were analyzed using a two-sample *t* test and a rank-sum analysis for parametric and nonparametric values, respectively. Correlations between continuous variables were obtained using Pearson's correlation coefficient (*r*_p_) for normal data and Spearman's rank correlation coefficient (*r*_s_) for nonnormal data. *P* values of less than 0.05 were considered to be statistically significant.

## 3. Results

### 3.1. Donor-Related Factors

The mean age of the donors in the foreign group was significantly higher than domestic group (Table 2) (*P* = 0.000), probably due to domestic donors mainly consisting of young and middle-aged volunteers. The mean P-O time was significantly longer in the foreign group than in the domestic group (Table 2) (*P* = 0.000) as a result of the long transportation distance that was required. The mean D-P time was, on the contrary, significantly shorter in the foreign group than in the domestic group (Table 2) (*P* = 0.000) as a result of the long transportation distance that was required. Corneal endothelial cell density (ECD) was lower in the foreign group (2204 ± 242 versus 3048 ± 277 mm^2^, *P* = 0.000), probably because of age differences in endothelial density between foreign donors and domestic donors or racial differences in endothelial density between white and Chinese populations.

### 3.2. Clinical Outcomes

Age, original disease, incidence of high-risk cases, and postoperative medication use were similar in both groups (Table 1). The most common cause of PKP was infectious keratitis (corneal ulcers) (53.7%) including bacterial keratitis (3.7%), viral keratitis (27.8%), and fungal keratitis (22.2%), followed by corneal leukoma (22.2%).

### 3.3. Prognosis of Infectious Keratitis

In foreign infectious keratitis group, 19 were cured (65.6%), 7 were improved (24.1%), and 3 were ineffective (10.3%), while in domestic infectious keratitis group, 17 were cured (58.6%), 8 were improved (27.6%), and 4 were ineffective (13.8%). No significant difference with respect to the surgical outcome was observed between the two groups (Table 3) (*P* = 0.687).

Recurrent fungal corneal ulcers were 3 and 4 cases in the foreign and domestic groups, respectively. Annular ulcers and perforate were observed at the edge of the graft in these 7 patients. Of these patients, 1 case in foreign donor group underwent repeated PKP and infection was controlled, whereas the others underwent eye enucleation as a result of entophthalmia.

### 3.4. BCVA and Neovascularization by Final Follow-Up

As shown in Table 4, the average BCVA by final follow-up were 1.28 ± 0.93 and 1.27 ± 1.04 in the foreign and domestic groups, respectively. No statistically significant difference between the two groups was observed (*P* = 0.553). However, postoperative BCVA in the two groups was all significantly higher than preoperatively value (all *P* < 0.05). When patients were further classified into infectious keratitis group and noninfectious keratitis group according to the type of infection, the average BCVA in foreign infectious keratitis group was significantly higher (*P* = 0.003), compared to the foreign noninfectious keratitis group. Similar results were observed within different subgroups in domestic donor group (*P* = 0.048). Overall, the BCVA in noninfectious keratitis group was better compared to infectious keratitis group.

In terms of neovascularization, no difference was observed between the foreign and domestic group (*P* = 0.811). After subdivided into different subgroup according to the type of infection, the neovascularization in foreign infectious keratitis group was also higher (*P* = 0.015) than the foreign noninfectious keratitis group. Similar results were observed within different subgroups in domestic donor group (*P* = 0.030).

Overall, the BCVA and neovascularization in noninfectious keratitis group were better compared to infectious keratitis group.

### 3.5. Corneal Graft Thickness and Corneal Endothelial Cell Change

We assessed corneal graft thickness at 1, 3, 6, and 12 months postoperatively in each group. We found that the graft thickness in the foreign group was higher compared to the domestic group (*t* = 16.17, *P* = 0.00) at 1 month postoperatively, but not at 3, 6, and 12 months postoperatively (Table 5).

**Figure 1:**
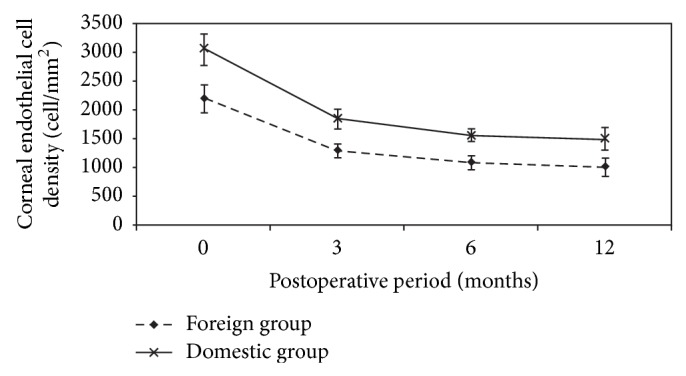
Corneal endothelial cell density (cell/mm^2^) changes in foreign and domestic donor groups before surgery and at 3, 6, and 12 months postoperatively.

We also assessed corneal endothelial cell density before surgery and at 3, 6, and 12 months postoperatively in each group. The average corneal endothelial cell density of donor corneas before surgery and at 3, 6, and 12 months after PKP was 2204 ± 242, 1309 ± 119, 1098 ± 121, and 1025 ± 147 cells/mm^2^ in the foreign group and 3048 ± 277, 1860 ± 165, 1573 ± 116, and 1508 ± 192 cells/mm^2^ in the domestic group, respectively. The corneal ECD in the domestic group was significantly higher than the foreign group at 3, 6, and 12 months postoperatively (Figure 1) (all *P* < 0.05). Postoperative corneal ECD in the two groups was also significantly lower than preoperatively value (all *P* < 0.05).

### 3.6. Graft Survival

**Figure 2:**
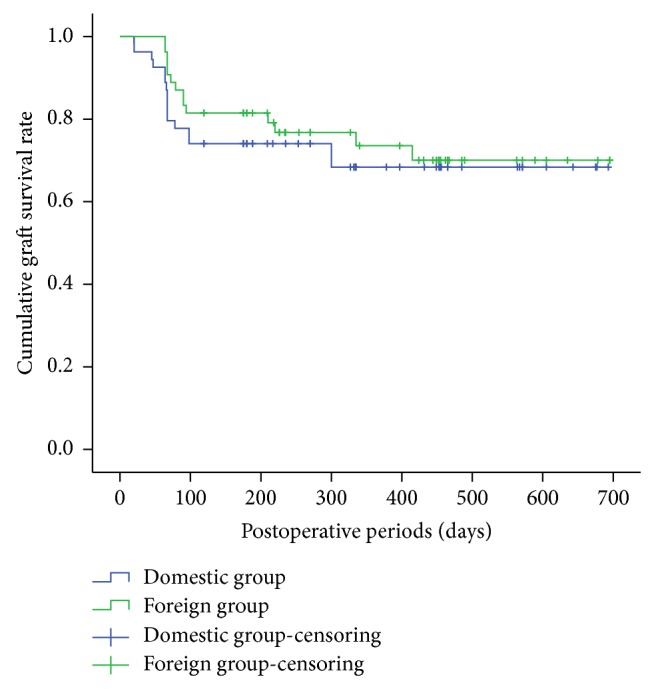
Kaplan-Meyer analysis of graft survival in the foreign (solid line) and domestic (dotted line) donor groups.

**Figure 3:**
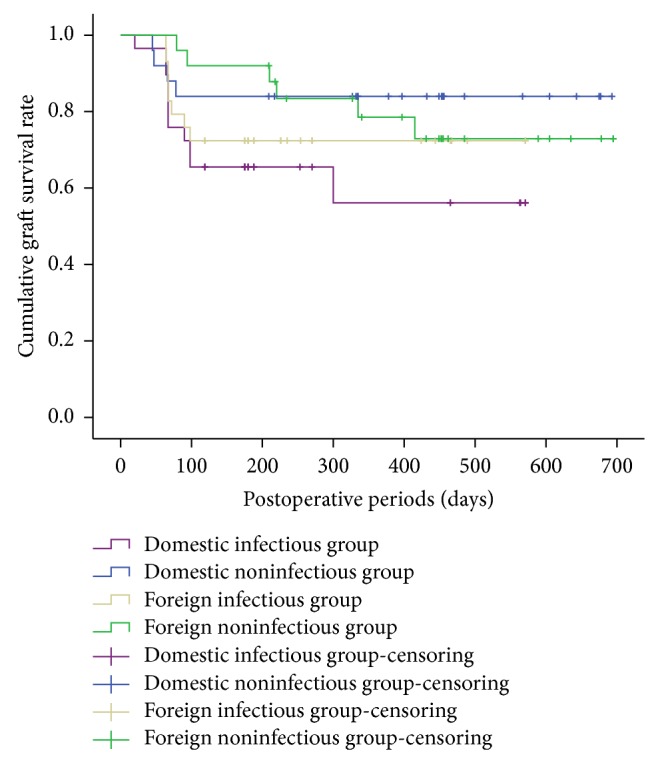
Kaplan-Meyer analysis of graft survival in the infectious and noninfectious subgroups within the foreign and domestic donor groups.

Overall, 84 of 108 corneas (77.8%) were clear at the time of final follow-up, with a mean follow-up period of 384 days. Kaplan-Meyer analysis demonstrated that the incidence of clear grafts was not significantly different between the two groups by final follow-up (Figure 2) (*P* = 0.672). However, when patients were further classified into infectious keratitis group and noninfectious keratitis group according to the type of infection, Kaplan-Meyer analysis showed significant difference with respect to the incidence of clear grafts within the two subgroups in the foreign donors (Figure 3) (*P* = 0.048).

At the end of the follow-up period, 12 (2 were primary graft failure) of patients in the foreign donor group and 9 of patients in the domestic group developed corneal graft decompensation. Of the 21 patients with corneal graft decompensation, 19 were caused by endothelial cell decompensation due to immunologic rejection except for the primary graft failure. Spearman analysis showed that the incidence of corneal graft decompensation was statistically correlated with immunologic rejection (*r* = 0.64, *P* = 0.021) and secondary glaucoma (*r* = 0.48, *P* = 0.033) (Table 6). Conversely, no correlation was observed between the incidence of corneal graft decompensation and source of graft, graft corneal epithelial abnormalities, and postoperative graft infection (all *P* > 0.05).

### 3.7. Postoperative Complications

#### 3.7.1. Primary Graft Failure

Primary graft failure, defined as persistent corneal edema starting immediately after surgery, was observed in two eyes (1.7%) in the foreign group (Table 7) (*P* = 0.153). This might be due to limited number of samples.

#### 3.7.2. Immunologic Rejection

The rate of immunologic rejection was comparable in both groups throughout the observation period. By the end of examination period, immunologic rejection was observed in 18 (33.3%) and 23 (42.6%) patients in the foreign and domestic groups, respectively (Table 7) (*P* = 0.322). These patients were treated with cyclosporine eye drops combined with prednisolone acetate eye drops or meprednisone by intravenous injection. After active antirejection treatment, 8 out of 18 in the foreign group and 6 out of 23 patients in the domestic group recovered to clear, whereas the others developed corneal graft decompensation.

#### 3.7.3. Corneal Epithelial Abnormalities

Corneal epithelial abnormalities with signs of corneal epithelial defects, mild edema, and epithelial keratinization developed postoperatively in 7 (13%) and 1 (1.8%) eyes in the foreign and domestic groups, respectively (*P* = 0.03) (Table 7). Persistent corneal epithelial defects with a time of duration of 2–8 weeks were seen in 5 cases of foreign donors postoperatively. The healed corneal epithelium showed signs of corneal epithelial dysplasia, mild edema, and epithelial keratinization.

#### 3.7.4. Secondary Corneal Infection

Positive cultures for the donor corneal rim were 2.4% and 5% in the foreign and domestic groups, respectively (*P* = 0.553) (Table 7). Positive cultures for the donor preservation medium were observed in 1 case in the domestic group. No postoperative infections were observed in the two groups.

#### 3.7.5. Secondary Glaucoma and Ocular Hypotension

Secondary glaucoma developed postoperatively in 12 (22.2%) and 14 (25.9%) eyes in the foreign and domestic groups, respectively (*P* = 0.653) (Table 7). These glaucoma patients were treated by trabeculectomy, endoscopic cyclophotocoagulation, and Ahmed glaucoma valve implant, and thereafter normal intraocular pressure was obtained from every patient. None of the patients in the two groups developed secondary ocular hypotension and eyeball atrophy.

## 4. Discussion

The shortage of donor corneas is a serious problem in China. The number of patients registered in eye banks is more than 2,000,000 whereas the annual number of transplants is approximately 3,000. Thus, most patients in small cities or remote rural areas must wait for several years before a cornea becomes available. Many of the patients in our series had been waiting for surgery for more than a year before coming to our institute. This long waiting time not only leads to significant socioeconomic and psychologic loss, but the surgical prognosis of many patients actually worsens, as in cases of progressive disease such as fungal cornea ulcer. Increasing the domestic supply of corneas is vital in overcoming the supply problem. However, despite the continuous efforts of social propaganda, the number of corneal donations remains insufficient to permit surgery to be performed within a reasonable period of time. The reasons for the continuous shortage in donors appear to be multifactorial in China, and a traditional cultures and religious faith are probably the largest cause. Shortages in donor corneas are also present in other Asian countries and Africa. The United States, however, has an exceptionally successful eye banking program. In 2001, US eye banks collected 92,578 donor corneas, but only 54.9% of them were used for corneal transplants. The Eye Bank Association of America provides the surplus donor corneas to various countries in South and Central America, the Middle East, Europe, and Asia. In 2001, 13,497 corneas (14.6% of the total donor corneas) were transported to countries outside the United States (Eye Bank Association of America, 2001 Eye Banking Statistical Report). Although using foreign corneas can alleviate the shortage in donor corneas for corneal transplantation, there have been concerns about the safety and efficacy of corneal transplantations with foreign donor corneas.

In the present study, the POT time differed between the foreign and domestic groups, reflecting the longer time required for transportation from the United States to China. However, our results suggest that this longer preservation time did not influence surgical outcome. The mean age of the foreign donors (66.3 years) in the present study was older than the average age of domestic donors (27.8 years). However, the clinical outcomes such as vision recovery and graft survival of our study were comparable in the two groups. This finding is consistent with the previous study reported by Cornea Donor Study Investigator Group which said that five-year graft survival for cornea transplants at moderate risk for failure is similar using corneas from donors ⩾ 66.0 years and donors < 66.0 years [7]. They also found that there was not a significant relationship between donor age and graft survival (*P* = 0.11).

Corneal thickness is one of the most important indicators for corneal health. In the present study, we assessed corneal graft thickness at 1, 3, 6, and 12 months postoperatively in each group. We found that the graft thickness in the foreign group was significantly higher compared to the domestic group at 1 month postoperatively, but not at 3, 6, and 12 months postoperatively. Our results suggest that the recovery rate of endothelial cells in foreign grafts is much lower compared to the domestic grafts which show a clinical symptom of continuous graft edema. This may be explained by the longer preservation time, longer time required for transportation from US to China. Another factor was that endothelial cell loss is higher in older donor corneal tissues [7]. During the process, series of biochemical reaction occurred in graft endothelial cells which affect the cell function. Postoperative follow up outcomes showed that the corneal thickness and clarify was similar in the two groups. Thus, corneal transplantations using foreign corneas are safe and effective.

Considering the control rate of the infectious keratitis, 26 (89.7%) in foreign infectious keratitis group and 25 cases (86.2%) in domestic infectious keratitis group were therapeutic effective. No significant difference with respect to the therapeutic outcome was observed between the two groups. The findings above suggest that corneal transplantations using foreign donor corneas have a similar effective as those using domestic donor corneas.

Our study showed that the average BCVA after PKP in the foreign and domestic groups was all significantly higher than preoperatively value and the BCVA in noninfectious keratitis group was better compared to infectious keratitis group. But when compared between the foreign and domestic group, no statistically significant difference with respect to the postoperative BCVA was observed. This may associated with the smaller graft diameter, low risk of recipient neovascularization, and low incidence of immunological rejection in the noninfectious keratitis group, whereas in the infectious group, to thoroughly remove the lesion of the original disease, graft with a relatively larger diameter was often used. Graft with a lager diameter and the inflammation of graft bed was associated with the high incidence of immunological rejection after PKP [9–11].

In our study, no statistically significant difference with respect to primary graft failure between foreign and domestic groups was observed. This may be due to small number of samples. And thus further study with a larger number of samples is still needed to confirm our results.

The cornea is commonly thought to be immunologically privileged as blood vessels and lymphatics are absent [12]. Therefore, corneal transplantation has become one of the most successful surgery techniques in organ transplantation. However, the immune privilege of the cornea is a relative concept of allograft rejection compared with the transplantation of other organs [13]. Thus, immunological rejection remains the primary causes of PKP, accounting for 33%–60% [12]. In this study, the incidence of immunologic rejection was 18 (33.3%) in the foreign group and 23 (42.6%) patients in domestic groups, which was similar to the previous report [12]. Despite the existence of difference with respect to the human HLA antigens and ABO blood type between the two groups, the rate of immunological rejection in foreign group was not higher than the domestic group (*P* > 0.05). Firstly, the primary cause can be attributed to immune privilege of the cornea. Dunn et al. suggested that ABO donor-recipient incompatibility was not associated with graft failure due to any cause including graft failure due to rejection or with the occurrence of a rejection episode. According to their report, the five-year cumulative incidence of graft failure due to rejection was 6% for recipients with ABO recipient-donor compatibility and 4% for those with ABO incompatibility (hazard ratio 0.65, 95% confidence interval 0.33 to 1.25, *P* = 0.20). The five-year incidence for a definite rejection episode, irrespective of whether graft failure ultimately occurred, was 12% for ABO compatible compared with 8% for ABO incompatible cases (*P* = 0.09). Therefore, ABO match was unnecessary because endothelial cell loss in graft was comparable [2, 14]. Secondary, the reduced antigen of the low temperature preserved corneas was another affecting factor [15]. Nguyen et al. [16] indicated that graft failure rate of long-term preserved donor corneas was lower compared to short-term-preserved corneas when PKP was performed. The findings of our study showed that the rate of immunologic rejection and graft clarify were comparable in both groups throughout the follow-up period.

Our results also showed that 12 (22.2%) in the foreign group and 14 (25.9%) patients in the domestic group progressed to secondary glaucoma postoperatively (*P* = 0.653). This is in accordance with a previous report [17]. The harm of secondary glaucoma was secondary to the immunological rejection, which usually resulting in opaque grafts. Seitz et al. [17] suggested that the incidence of secondary glaucoma after PKP mainly depended on the preoperative original disease and associated with complexity of microsurgical technique, careful suturing, and peripheral iridotomy. In our study, factors affecting the incidence of secondary glaucoma after PKP may include (1) long-term use of corticosteroid drugs which was used to prevent immunological rejection and control the inflammatory response; (2) primary angle-closure which was associated with trabecular meshwork damage and peripheral anterior synechiae; (3) surgical factors: complex intraocular surgery and severe postoperative inflammatory response. Besides, our study showed that there was no significant difference with respect to the incidence of secondary glaucoma postoperatively between the two groups, which is consistent with the literature reported by Shimazaki et al. [1].

In our study, there were abnormal corneal epithelial with signs of corneal epithelial defects, mild edema, and epithelial keratinization in 7 (13%) and 1 (1.8%) cases in the foreign and domestic groups, respectively (*P* = 0.03). Persistent corneal epithelial defects with a time of duration of 2–8 weeks were observed in 5 cases of foreign donors postoperatively. The healed corneal epithelium showed signs of dysplasia, mild edema, and keratinization. These may be the cause of longer time required for preservation and transportation from the United States to China. During the process, it may produce a series of biochemical reaction, resulting in the epithelial dysplasia or long time to healing. Polyak et al. [18] reported that low-temperature preserved corneal epithelial cell in a frozen state have a relatively a slightly lower tolerability for freezing injury; however, the proliferation ability did not show any obvious difference compared to the fresh corneal epithelial cell. Therefore, to avoid postoperative graft abnormalities, donor corneas with a shorter preservation time should be given priority for PKP.

Our study showed that positive cultures for the donor corneal rim were 2.4% and 5% in the foreign and domestic groups, respectively (*P* = 0.553). There was only 1 case of positive cultures for the donor preservation medium in the domestic group. Besides, there were also no secondary graft infections postoperatively in the two groups. Our results are consistent with the previous report [1]. These findings suggest that longer preservation time did not influence surgical outcome.

## 5. Conclusion

Despite the fact that there exists minor difference between the foreign and domestic donor corneas, the efficacy and safety of corneal transplantations using foreign donor corneas or domestic donor corneas was similar in terms of therapeutic outcome, BCVA, neovascularization, primary graft failure, graft survival, and postoperative complications such as immunologic rejection, graft infection, and secondary glaucoma. Organ-sharing programs may be of great benefit to patients in countries with shortages of organs for transplantation.

## Figures and Tables

**Table 1:** Patients' characteristics.

	Foreign group	Domestic group	*P *value
Number of patients	54	54	
Mean age (years)	54.2 ± 11.2	53.8 ± 15.0	0.876
Sex (M : F)	40 : 14	36 : 18	0.399
Follow-up period (days)	301 ± 198	326 ± 213	0.529
BCVA (log MAR)	1.51 ± 0.59	1.46 ± 0.55	0.650
Original diseases (*n*, %)			
Infectious keratitis (Ulcer)			
Bacteria	2 (3.7)	2 (3.7)	
Viral	15 (27.8)	15 (27.8)	
Fungal	12 (22.2)	12 (22.2)	
Leukoma	12 (22.2)	12 (22.2)	
Bullous keratopathy	3 (5.6)	3 (5.6)	
Corneal degeneration	4 (7.4)	4 (7.4)	
Keratoconus	2 (3.7)	2 (3.7)	
Second operation	4 (7.4)	4 (7.4)	1.000
Preoperative glaucoma (%)	0 (0)	0 (0)	
Neovascularization (%)			
0	32 (59.3)	32 (59.3)	
+	20 (37.0)	18 (33.3)	
++	2 (3.7)	4 (7.4)	
+++	0 (0)	0 (0)	
++++	0 (0)	0 (0)	0.879

**Table 2:** Donor-related factors.

	Foreign group (*n* = 54)	Domestic group (*n* = 54)	*P* value
Mean age (years)	66.3 ± 8.7	27.8 ± 11.3	=0.000
DPT (hours)	6.3 ± 2.2	10.4 ± 5.3	=0.001
POT (hours)	125.4 ± 36.7	23.6 ± 8.9	=0.000
ECD (cell/mm^2^)	2204 ± 242	3048 ± 277	=0.000

DPT: death-to-preservation time; POT: preservation-to-operation time; ECD: endothelial cell density.

**Table 3:** Surgical outcome of infectious keratitis between the two groups.

Outcome	Foreign infectious group	Domestic infectious group	*P* value
Effectiveness			0.687
Cure	19 (65.6%)	17 (58.6%)	
Improvement	7 (24.1%)	8 (27.6%)	
Ineffectiveness	3 (10.3%)	4 (13.8%)	

Therapeutic effectiveness was achieved if infectious keratitis (corneal ulcers) was cured or improved.

**Table 4:** BCVA and neovascularization by final follow-up in different groups.

Groups	Post-BCVA (log MAR)	Neovascularization				
		−	+	++	+++	++++
Foreign group	1.28 ± 0.93	12	20	16	4	2
Noninfectious group	0.77 ± 0.49	8	11	5	1	0
Infectious group	1.76 ± 1.00^*^	4	9	11	3	2
Domestic group	1.27 ± 1.04	16	16	14	4	4
Noninfectious group	0.80 ± 0.53	10	8	6	1	0
Infectious group	1.49 ± 1.20	6	8	8	3	4

^*^ *P* < 0.05 versus noninfectious group; BCVA: best corrected visual acuity.

**Table 5:** Postoperative graft thickness (mm) in foreign and domestic donor groups at follow-up.

Postoperative follow-up (months)	Foreign group (*n* = 54)	Domestic group (*n* = 54)	*t*	*P* value
1	0.84 ± 0.08	0.62 ± 0.06	16.17	0.000^*^
3	0.59 ± 0.08	0.57 ± 0.06	1.47	0.145
6	0.56 ± 0.06	0.54 ± 0.04	2.04	0.051
12	0.54 ± 0.05	0.54 ± 0.04	0.00	1.000

^*^ *P* < 0.001.

**Table 6:** Spearman analysis for the factors affecting postoperative graft decompensation.

Index	Graft decompensation	
	*r*	*P* value
Postoperative rejection	0.64	0.021
Postoperative secondary glaucoma	0.48	0.033
Source of graft (foreign/domestic)	0.15	0.767
Epithelial abnormalities	0.16	0.564
Graft infection	0.24	0.412

**Table 7:** Postoperative complications and contamination of the corneal rim and preservation medium in foreign and domestic donor groups.

	Foreign group (%)	Domestic group (%)	*P *value
Primary graft failure	2 (3.7)	0 (0)	0.153
Immunologic rejection	18 (33.3)	23 (42.6)	0.322
Secondary glaucoma	12 (22.2)	14 (25.9)	0.653
Corneal epithelial abnormalities	7 (13.0)	1 (1.8)	0.03
Secondary corneal infection	0 (0)	0 (0)	
Positive culture for bacteria			
Donor rim	1/42 (2.4)	2/40 (5)	0.553
Preservation medium	0/42 (0)	1/40 (2.5)	0.98
